# Association of preoperative serum 25-hydroxyvitamin D levels with deep vein thrombosis after total knee arthroplasty: a hypothesis-generating analysis of coagulation function mechanisms

**DOI:** 10.3389/fsurg.2026.1756540

**Published:** 2026-08-27

**Authors:** He Shang, Jun Li, Tao Ma, Yuan Wei, Jinpeng Liang, Yumei Ding, Xing He, Biao Ma, Yaxing Ma, Ruoyu Wang, Yi Wang, Desheng Chen

**Affiliations:** 1 Third Clinical Medical College of Ningxia Medical University, People’s Hospital of Ningxia Hui Autonomous Region, Yinchuan, China; 2 Department of Joint Surgery, Ningxia Hui Autonomous Region People’s Hospital (Affiliated Hospital of Ningxia Medical University), Yinchuan, China; 3 Department of Pediatrics, Affiliated Hospital of Xuzhou Medical University, Xuzhou, China

**Keywords:** 25-hydroxyvitamin D, coagulation function, deep vein thrombosis, hypothesis-generating, total knee arthroplasty

## Abstract

**Objective:**

This hypothesis-generating study aimed to explore the association of preoperative serum 25-hydroxyvitamin D [25(OH)D] levels with deep vein thrombosis (DVT) risk after total knee arthroplasty (TKA) and clarify the potential coagulation-related mechanisms.

**Methods:**

A total of 193 patients undergoing primary unilateral TKA in a single center were stratified into 25(OH)D deficiency (<20 ng/mL, *n* = 106), insufficiency (20–29 ng/mL, *n* = 47), and sufficiency (≥30 ng/mL, *n* = 40) groups. Postoperative DVT (detected via day 5–7 venous ultrasonography) and coagulation indices [D-dimer, prothrombin time (PT)] were compared across groups. Univariate and multivariate logistic regression were used to screen DVT-related factors, and Firth's penalized logistic regression was applied for a sparse-data sensitivity analysis.

**Results:**

The overall postoperative DVT incidence was 13.5% (26/193). A bivariate analysis revealed a markedly higher DVT rate in the deficiency group (18.9%) than in the sufficiency group (2.5%), with a crude 2 × 2 odds ratio (OR) of 9.070 [95% confidence interval (CI): 1.198–68.542, *P* = 0.015]; this estimate was unstable due to only one DVT case in the sufficiency group. To provide a more stable estimate, a secondary binary logistic regression model (deficiency vs. non-deficiency) was performed, which identified preoperative 25(OH)D deficiency as significantly correlated with postoperative DVT (OR = 3.140, 95% CI: 1.200–8.212, *P* = 0.020). After analysis of covariance adjustment for baseline levels, the deficiency group maintained significantly higher postoperative D-dimer and shorter PT than the sufficiency group (both *P* < 0.001). A univariate analysis identified 25(OH)D deficiency and elevated postoperative D-dimer as DVT-related risk factors. In the secondary exploratory model adjusting for postoperative D-dimer (a potential mediator), the association between vitamin D deficiency and DVT was attenuated (OR = 2.576, 95% CI: 0.941–7.049, *P* = 0.065), indicating a possible mediating effect rather than confounding bias. Firth's sensitivity analysis on the binary model further verified the robustness of this finding (OR = 2.918, 95% CI: 1.142–7.456, *P* = 0.025).

**Conclusion:**

Preoperative 25(OH)D deficiency is associated with elevated postoperative DVT risk after TKA in univariate analyses. Abnormal postoperative D-dimer and PT levels suggest that vitamin D status may modulate coagulation-fibrinolysis balance. The attenuated association after D-dimer adjustment supports a potential mediating mechanism. As a hypothesis-generating analysis, these preliminary findings require further validation in large-scale prospective studies to confirm the independent predictive value of preoperative vitamin D deficiency for post-TKA DVT.

## 1 Introduction

Total knee arthroplasty (TKA) is a common surgical procedure for treating end-stage knee osteoarthritis, significantly improving joint function and quality of life (1). However, postoperative deep vein thrombosis (DVT) is a serious complication with a relatively high incidence in orthopedic surgeries; it often progresses to pulmonary embolism (PE), posing life-threatening risks (2). Observational studies confirm that venous thromboembolism (VTE) is a frequent complication of major orthopedic procedures such as hip fracture repair, total hip arthroplasty, and TKA (3). Although prophylactic strategies (e.g., anticoagulants like low-molecular-weight heparins) reduce this risk (4), the 90-day risk of VTE after such surgery remains approximately 1.0%–1.5% despite standardized prophylaxis (5). Current clinical practice involves combined mechanical (e.g., intermittent pneumatic compression) and pharmacological prophylaxis (6). Nevertheless, some patients still develop DVT, partly because individual risk factors are not fully identified (7).

Serum 25-hydroxyvitamin D [25(OH)D] is the primary indicator of vitamin D nutritional status. Evidence suggests that vitamin D deficiency [VDD, commonly defined as serum 25(OH)D < 20 ng/mL] may contribute to thrombogenesis through alterations in coagulation and endothelial function or inflammatory pathways (8, 9). Large cohort studies, including retrospective analyses using databases like TriNetX, indicate that VDD is independently associated with an increased risk of VTE, exhibiting a dose-dependent relationship [risk elevates as serum 25(OH) D decreases] (10). This association extends to DVT and PE incidence rates and correlates with higher patient mortality and increased healthcare utilization.

However, despite the volume of observational data, significant controversy exists. Systematic reviews have not established a definitive mechanistic link between lower vitamin D levels and prothrombotic parameters (e.g., in hemostasis and fibrinolysis systems), despite observational data suggesting an association with increased thrombotic event risk (11). There is a lack of high-quality randomized controlled trial evidence supporting a specific antithrombotic effect or confirming a causal pathway (12); subgroup analyses have not shown consistent molecular associations, and mechanistic findings remain conflicting (13). Specifically, within the TKA population, the impact of VDD on postoperative DVT risk is inconclusive: some studies (e.g., matched cohort designs) support preoperative VDD as an independent risk factor for VTE (10), while other systematic evidence has not confirmed a direct association between vitamin D deficiency and thrombotic predisposition (14).

Given these controversies, this retrospective study of 193 patients who underwent TKA aims to investigate the correlation between preoperative 25(OH)D levels and the incidence of postoperative DVT, as well as coagulation parameters such as D-dimer. Given the modest sample size, this study is explicitly intended to generate hypotheses for future larger-scale investigations and not to provide definitive answers.

## 2 Materials and methods

### 2.1 Research subjects

A single-center retrospective cohort study was conducted. A total of 193 consecutive patients who underwent primary unilateral TKA in the People's Hospital of Ningxia Hui Autonomous Region from January 2020 to September 2023 were enrolled. The inclusion criteria were: (1) age ≥50 years; (2) diagnosis of knee osteoarthritis or rheumatoid arthritis confirmed by X-ray and clinical assessment, with an indication for TKA; (3) serum 25(OH)D concentration measured using chemiluminescence immunoassay within 1 week preoperatively (7); (4) a bilateral lower limb compression venous ultrasound performed between postoperative days 5 and 7 to assess for DVT, with interpretations independently conducted by at least two blinded radiologists (15); and (5) complete collection of baseline data, including age, sex, body mass index (BMI), comorbidities, surgical records, and laboratory parameters such as prothrombin time (PT) and D-dimer levels.

The exclusion criteria were: (1) preoperative imaging-confirmed DVT (all patients underwent a preoperative bilateral ultrasound to exclude pre-existing DVT), PE, or a history of VTE; (2) long-term use (≥3 months) of anticoagulants (e.g., warfarin and rivaroxaban) or antiplatelet agents (e.g., aspirin and clopidogrel) to mitigate confounding effects on coagulation (6); (3) coagulation disorders (international normalized ratio, INR >1.5), hematologic diseases, moderate-to-severe hepatic or renal impairment (defined as Child-Pugh grade B or C), or active malignancies; (4) a history of vitamin D or calcium supplementation within 3 months preoperatively to avoid alterations in baseline 25(OH)D status (10); (5) pregnancy or lactation; and (6) concurrent acute infections, traumatic injuries, or systemic inflammatory conditions.

### 2.2 Grouping criteria

For research classification purposes, patients were stratified into three groups based on preoperative serum 25(OH)D levels, according to the established clinical practice guidelines from the Endocrine Society: deficiency group (<20 ng/mL), insufficiency group (20–29 ng/mL), and sufficiency group (≥30 ng/mL) (16). Although we acknowledge that the more recent 2024 Endocrine Society guideline recommends against routine screening in healthy asymptomatic adults, these specific thresholds are used here strictly for research classification to ensure comparability with a large body of existing literature (17).

### 2.3 Observation indicators and detection methods

Based on comprehensive patient baseline data extraction from the hospital electronic medical record system, the following information was collected: demographic characteristics (age, sex, height, and weight, with BMI calculated), clinical features (duration of hospitalization, operative side), and comorbidities. Diabetes mellitus was defined according to established clinical criteria. Hypertension was defined based on standard clinical guidelines. Cardiovascular disease encompassed coronary artery disease, heart failure, and arrhythmias.

Venous blood sampling was performed according to a standardized protocol. Fasting venous blood (5 mL) was collected within 1 week preoperatively. Serum was separated by centrifugation at 3,000 rpm for 10 min. Serum 25(OH)D concentrations were measured using an electrochemiluminescence immunoassay on a Roche Cobas e601 automated analyzer with manufacturer-matched reagents. Vitamin D status was categorized as follows: sufficient (≥30 ng/mL), insufficient (20–29 ng/mL), or deficient (<20 ng/mL).

Additional fasting venous blood samples (3 mL each) were collected 1 day preoperatively and on postoperative day 5. D-dimer levels were quantified by using an immunoturbidimetric assay, and PT was measured using a coagulometric method on a Siemens XPT automated coagulation analyzer. Normal reference ranges were defined as 0–0.5 mg/L for D-dimer and 11–14 s for PT. Blood samples for postoperative D-dimer and PT were collected on postoperative day 5, which preceded the confirmatory ultrasound in the majority of cases. All procedures strictly adhered to the manufacturer's instructions for the instruments and reagent kits.

A bilateral lower-extremity venous ultrasonography (encompassing the iliac, femoral, popliteal, anterior tibial, and posterior tibial veins) was performed by two experienced sonologists, independently, between postoperative days 5 and 7. The diagnostic criteria for DVT included: (1) visualization of intraluminal solid echogenicity with non-compressibility of the vein upon transducer pressure; (2) interruption of color flow signals or the presence of a filling defect; and (3) absence of flow signal or abnormal flow velocity on a pulsed-wave Doppler examination. In cases of diagnostic disagreement between the two initial sonologists, a final determination was made by a third senior ultrasonologist.

### 2.4 Surgery and perioperative management

A standardized perioperative protocol was implemented for all surgeries performed by a consistent team of orthopedic surgeons, utilizing combined spinal anesthesia and sciatic nerve block. Intraoperatively, a tourniquet was applied with pressure set to systolic blood pressure plus 50 mmHg, with a single-use duration ≤90 min. Postoperatively, drains were routinely placed and removed within 24–48 h. Perioperative thromboprophylaxis included a subcutaneous injection of low-molecular-weight heparin calcium at a fixed dose of 4,100 IU once daily (not weight-adjusted) initiated 12 h after surgery and continued until discharge (extended prophylaxis beyond discharge was not routinely administered); intermittent pneumatic compression therapy for both lower extremities administered twice daily starting 6 h postoperatively; and guided ankle pump exercises with knee flexion-extension training commencing 24 h postoperatively under rehabilitation guidance.

### 2.5 Statistical methods

Statistical analyses were conducted using SPSS software version 26.0 (IBM Corp., Armonk, NY, USA), Python version 3.10 (Python Software Foundation, Delaware, USA), and R version 4.x (R Foundation for Statistical Computing, Vienna, Austria) for the Firth penalized logistic regression sensitivity analysis. Continuous variables were expressed as mean ± standard deviation, whereas categorical data were presented as numbers (%). For comparisons among multiple groups, one-way analysis of variance (ANOVA) was applied, followed by least significant difference *post hoc* tests for pairwise comparisons where appropriate. For postoperative coagulation parameters, analysis of covariance (ANCOVA) was used to adjust for baseline (preoperative) values. Within-group comparisons before and after surgery were assessed using paired Student's *t*-tests. Between-group comparisons for categorical variables were performed using the chi-square test or Fisher's exact test if expected frequencies were below 5.

For the primary analysis, univariate logistic regression was employed to screen potential factors associated with DVT occurrence using a correctly specified three-level categorical variable for vitamin D status (deficiency, insufficiency, sufficiency) with sufficiency as the reference. Because of the limited number of DVT events (*n* = 26), and specifically the sparse cell in the sufficiency group, a secondary binary logistic regression model (deficiency vs. non-deficiency) was also employed to provide a more stable estimate. Variables with *P* < 0.05 in the univariate analysis were subsequently considered for a multivariate logistic regression model. The multivariate model included only binary vitamin D status (deficiency vs. non-deficiency) and postoperative D-dimer as a continuous variable, with the understanding that D-dimer is likely a mediator rather than a confounder. Odds ratios (ORs) and 95% confidence intervals (CI) were calculated. Statistical significance was defined as *P* < 0.05 for all tests. As a sensitivity analysis for sparse data (particularly the single DVT event in the sufficiency group), Firth's penalized logistic regression with profile-likelihood confidence intervals was performed on the binary model (deficiency vs. non-deficiency) using the logistf package in R. Given the exploratory, hypothesis-generating nature of this study, no adjustment for multiple comparisons was applied.

## 3 Results

### 3.1 Comparison of baseline characteristics among the three patient groups

Among 193 patients, vitamin D status was categorized as deficient (*n* = 106, 54.9%), insufficient (*n* = 47, 24.4%), or sufficient (*n* = 40, 20.7%). No statistically significant differences were observed among the groups in terms of age, BMI, hospital stay duration, sex distribution, prevalence of hypertension, prevalence of heart disease, or surgical laterality (all *P* > 0.05) (Table 1). However, a statistically significant difference in the prevalence of diabetes mellitus was noted, with the insufficient group exhibiting a higher rate of 21.3% compared with 7.5% in the deficient group (*χ*^2^ = 6.369, *P* = 0.041). This counterintuitive finding (lowest diabetes rate in the deficient group) may be attributed to chance or unmeasured confounding (e.g., dietary habits and medication use).

**Table 1:** Comparison of baseline characteristics among the three patient groups.

Variable	Deficiency group (*n* = 106)	Insufficiency group (*n* = 47)	Sufficiency group (*n* = 40)	Statistic	*P*-value
Age (years, mean ± SD)	66.46 ± 8.53	67.51 ± 7.96	68.12 ± 7.24	*F* = 0.700	0.498
BMI (kg/m^2^, mean ± SD)	25.71 ± 2.93	25.53 ± 2.86	25.35 ± 2.81	*F* = 0.237	0.789
Hospital stay (days, mean ± SD)	8.08 ± 2.10	7.79 ± 1.65	7.95 ± 1.52	*F* = 0.384	0.681
Male sex (*n*, %)	30 (28.3)	19 (40.4)	13 (32.5)	*χ*^2^ = 6.069	0.194
Diabetes (*n*, %)	8 (7.5)	10 (21.3)	7 (17.5)	χ^2^ = 6.369	0.041
Hypertension (*n*, %)	41 (38.7)	22 (46.8)	13 (32.5)	χ^2^ = 1.901	0.386
Heart disease (*n*, %)	8 (7.5)	7 (14.9)	6 (15.0)	χ^2^ = 2.695	0.260
Operated side (left/right, *n*)	54/52	29/18	18/22	χ^2^ = 2.598	0.273

### 3.2 Comparison of postoperative DVT incidence among the three patient groups

Of 193 surgical patients, 26 developed postoperative DVT, for an overall incidence of 13.5%. DVT incidence differed significantly among the three groups (*χ*^2^ = 7.102, *P* = 0.029), with the highest rate in the deficient group (18.9%) and the lowest in the sufficient group (2.5%). Among the 26 DVT events, 21 (80.8%) were distal (below-knee) and 5 (19.2%) were proximal (popliteal or femoral); all were asymptomatic at the time of screening. Pairwise comparisons (bivariate analysis) showed a significantly elevated unadjusted DVT risk in the deficient group vs. the sufficient group (unadjusted OR = 9.070; 95% CI: 1.198–68.542; *P* = 0.015) based on a 2 × 2 table; however, this estimate is based on a single DVT event in the sufficiency group and is therefore unstable. As a sensitivity analysis, assuming one additional DVT in the sufficiency group (i.e., two events out of 40) would change the OR to 4.42 (95% CI: 0.93–22.67). No significant differences were found between the deficient and insufficient groups or between the insufficient and sufficient groups (all *P* > 0.05) (Table 2).

**Table 2:** Comparison of postoperative deep vein thrombosis incidence among the three groups.

Group	Total number of cases	DVT occurrence (*n*, %)	No DVT (*n*)	Proportion of total DVT cases (%)
Deficient group	106	20 (18.9)	86	76.9
Insufficient group	47	5 (10.6)	42	19.2
Sufficient group	40	1 (2.5)	39	3.8
Total	193	26 (13.5)	167	100.0

### 3.3 Comparison of preoperative and postoperative coagulation function indicators among the three patient groups

Significant differences in preoperative day 1 plasma D-dimer levels and PT were observed among the three groups (all *P* < 0.05). The vitamin D deficiency group exhibited the shortest PT, while D-dimer levels were highest in the insufficiency group. By postoperative day 5, both parameters demonstrated significant alterations compared with preoperative values (all *P* < 0.001). Intergroup differences became more pronounced, as confirmed by ANOVA results (D-dimer: *F* = 13.991, *P* < 0.001; PT: *F* = 16.807, *P* < 0.001). After adjusting for baseline (preoperative) values using ANCOVA, the postoperative differences remained statistically significant (D-dimer: adjusted *F* = 11.234, *P* < 0.001; PT: adjusted *F* = 14.891, *P* < 0.001). Postoperative D-dimer levels revealed a hierarchical gradient: deficiency group > insufficiency group > sufficiency group. Conversely, PT exhibited an inverse gradient: deficiency group < insufficiency group < sufficiency group. Pairwise comparisons indicated statistically significant differences between the deficiency and sufficiency groups for both parameters postoperatively (all *P* < 0.001) (Table 3).

**Table 3:** Comparison of preoperative and postoperative coagulation function indicators among the three groups of patients (mean ± SD).

Indicator	Time point	Deficient group (*n* = 106)	Insufficient group (*n* = 47)	Sufficient group (*n* = 40)	*F*-value	*P*-value
D-dimer (mg/L)	Preoperative day 1	0.443 ± 0.347	0.517 ± 0.414	0.326 ± 0.156	3.552	0.031
	Postoperative day 5	2.572 ± 1.225	1.889 ± 0.911	1.556 ± 1.104	13.991	<0.001
PT (s)	Preoperative day 1	10.555 ± 0.899	10.945 ± 1.323	10.965 ± 1.200	3.274	0.040
	Postoperative day 5	11.212 ± 1.050	11.862 ± 1.249	12.397 ± 1.283	16.807	<0.001

### 3.4 Analysis of factors associated with postoperative DVT

#### 3.4.1 Univariate logistic regression analysis

A univariate logistic regression analysis was performed to screen potential associated factors. For the primary analysis, a correctly specified three-level model for vitamin D status (deficiency, insufficiency, and sufficiency) with sufficiency as the reference was used. The analysis demonstrated that preoperative vitamin D deficiency (OR = 9.070, 95% CI: 1.198–68.542, *P* = 0.015) and insufficiency (OR = 4.643, 95% CI: 0.517–41.731, *P* = 0.166) were associated with postoperative DVT, although the estimate for the deficiency group was highly unstable. Elevated postoperative D-dimer levels (OR = 1.414, 95% CI: 1.021–1.958, *P* = 0.037) were also significantly associated with postoperative DVT. Factors such as age, BMI, diabetes, and hypertension showed no significant association with DVT development (all *P* > 0.05). Because of the sparse cell in the sufficiency group, a secondary binary logistic regression model (deficiency vs. non-deficiency) was employed to provide a more stable estimate. This model identified preoperative 25(OH)D deficiency as a significant associated factor (OR = 3.140, 95% CI: 1.200–8.212, *P* = 0.020). As a sensitivity analysis using Firth's penalized logistic regression on the binary model (which addresses sparse-data bias), the OR was 2.918 (95% CI: 1.142–7.456, *P* = 0.025), consistent with the standard binary logistic regression result (Table 4).

**Table 4:** Univariate logistic regression analysis of the occurrence of postoperative DVT.

Variable	Regression coefficient	Standard error	Wald χ^2^	*P*-value	OR	95% CI
Three-level model (primary analysis)						
Preoperative 25(OH)D deficiency (vs. sufficiency)	2.205	1.026	4.617	0.032	9.070	1.198–68.542
Preoperative 25(OH)D insufficiency (vs. sufficiency)	1.536	1.119	1.884	0.170	4.643	0.517–41.731
Binary model (secondary, stable estimate)						
Preoperative 25(OH)D deficiency (vs. non-deficiency)	1.144	0.491	5.439	0.020	3.140	1.200–8.212
Other variables (from the binary model)						
Age (years)	−0.011	0.026	0.165	0.685	0.990	0.940–1.041
BMI (kg/m^2^)	−0.091	0.074	1.507	0.220	0.913	0.790–1.056
Diabetes (yes vs. no)	−0.151	0.655	0.053	0.818	0.860	0.238–3.104
Hypertension (yes vs. no)	−0.435	0.453	0.923	0.337	0.647	0.266–1.573
Heart disease (yes vs. no)	−1.224	1.047	1.366	0.242	0.294	0.038–2.290
Surgical side (left vs. right)	0.187	0.402	0.217	0.641	1.205	0.566–2.563
Postoperative D-dimer (mg/L)	0.346	0.166	4.342	0.037	1.414	1.021–1.958
Postoperative PT (s)	−0.012	0.171	0.005	0.943	0.988	0.707–1.380
Firth's penalized regression (on the binary model)						
Preoperative 25(OH)D deficiency (vs. non-deficiency)	–	–	–	0.025	2.918	1.142–7.456

The three-level model is the primary analysis. Because of the sparse cell (single DVT event in the sufficiency group), the binary model (deficiency vs. non-deficiency) is presented as a secondary and more stable estimate. Firth's penalized regression was applied to the binary model to further address sparse-data bias.

#### 3.4.2 Multivariate logistic regression analysis

In a secondary multivariate logistic regression model, we included binary vitamin D status (deficiency vs. non-deficiency, combining insufficiency and sufficiency) and postoperative D-dimer. This model was exploratory because D-dimer is likely a mediator rather than a confounder, and adjusting for it may attenuate the total effect of vitamin D on DVT. The results indicated that preoperative 25(OH)D deficiency was not a statistically significant factor associated with DVT in this secondary model (OR = 2.576, 95% CI: 0.941–7.049, *P* = 0.065). Postoperative D-dimer levels also showed no significant association with DVT occurrence in this model (*P* = 0.182) (Table 5). Diabetes was not included in the multivariate model because it was not significantly associated with DVT in the univariate analysis (*P* = 0.818), and adding it would have reduced the events-per-variable (EPV) ratio.

**Table 5:** Multivariable logistic regression analysis of postoperative DVT occurrence (secondary exploratory model).

Variable	Regression coefficient	Standard error	Wald χ^2^	*P*-value	OR	95% CI
Preoperative 25(OH)D deficiency (binary vs. non-deficiency)	0.946	0.514	3.395	0.065	2.576	0.941–7.049
Postoperative D-dimer (mg/L)	0.237	0.178	1.780	0.182	1.267	0.895–1.795
Constant	−3.040	0.549	30.625	<0.001	0.048	–

## 4 Discussion

### 4.1 Association between preoperative 25(OH)D levels and postoperative DVT following TKA (hypothesis-generating)

This study, utilizing real-world clinical data from 193 patients undergoing TKA, explored the association between preoperative 25(OH)D deficiency and postoperative DVT risk. The incidence of DVT was 18.9% in the deficiency group vs. 2.5% in the sufficiency group in the bivariate analysis. The primary univariate logistic regression, using a three-level model, identified 25(OH)D deficiency (OR = 9.070) and insufficiency (OR = 4.643) as associated factors, but these estimates were highly unstable. To obtain a more stable estimate, a binary model (deficiency vs. non-deficiency) was used, which confirmed 25(OH)D deficiency as a significant associated factor (OR = 3.140, 95% CI: 1.200–8.212, *P* = 0.020). This is consistent with findings from numerous international studies (18). For instance, Wang et al. (19) reported that individuals with 25(OH)D levels below 20 ng/mL had a 2.3-times higher risk of venous thromboembolism than those with levels ≥30 ng/mL (OR = 2.30, 95% CI: 1.12–4.72). Korkmaz et al. (20) showed significantly lower vitamin D levels in patients with lower-extremity DVT compared with controls.

However, the OR of 9.070 from the three-level model is based on only one DVT event in the sufficiency group, making it highly unstable. A sensitivity analysis assuming one additional DVT in the sufficiency group reduces the OR to 4.42 (95% CI: 0.93–22.67), illustrating the fragility of this estimate. Therefore, we emphasize the binary logistic regression OR of 3.140 as the more stable estimate.

In a secondary multivariate model adjusting for postoperative D-dimer (which lies on the causal pathway as a potential mediator), preoperative 25(OH)D deficiency did not achieve statistical significance (OR = 2.576, 95% CI: 0.941–7.049, *P* = 0.065). This attenuation is consistent with mediation rather than confounding and does not invalidate the observed univariate association. The events-per-variable ratio for this secondary model was 13 (26 events/two variables), which meets the recommended threshold of 10; therefore, the non-significant *P*-value (0.065) cannot be simply attributed to insufficient statistical power. However, the wide confidence interval (0.941–7.049) crossing 1.0 indicates considerable imprecision, and the point estimate (OR = 2.6) suggests a clinically meaningful effect that warrants further investigation in adequately powered studies. These findings should be interpreted as hypothesis-generating rather than confirmatory. Future large-scale prospective studies with adequate power are needed to validate whether preoperative 25(OH)D deficiency independently predicts DVT following TKA and to clarify the mediational role of coagulation markers.

### 4.2 Potential mechanisms by which vitamin D may affect postoperative DVT in TKA (speculative)

Based on the coagulation function data from this study and previous basic research, we speculate that vitamin D may participate in the development of postoperative DVT following TKA through several mechanisms, although these remain speculative given the observational nature of this study and the lack of direct biomarker measurements (e.g., inflammatory cytokines and endothelial markers).

#### 4.2.1 Regulation of the coagulation-fibrinolysis system balance

Disruptions in coagulation represent a central pathological mechanism underlying DVT formation after TKA. Our findings of significantly higher postoperative D-dimer levels and shorter PT in the vitamin D deficiency group provide clinical data consistent with the hypothesis of a hypercoagulable state. Elevated D-dimer indicates enhanced fibrin formation and degradation, a hallmark of hypercoagulability, while shorter PT suggests a potential hypercoagulable state. Studies demonstrate that serum 25(OH)D levels are markedly lower in individuals with DVT compared with controls, highlighting a relationship between vitamin D status and thrombotic events (20). In deficiency states, such disturbances may contribute to imbalances in coagulation and fibrinolysis, potentially exacerbating hypercoagulability and elevating thrombotic risk, as reflected in our coagulation parameter results.

#### 4.2.2 Protection of vascular endothelial function

Conversely, vitamin D sufficiency enhances endothelial function by upregulating endothelial nitric oxide synthase (eNOS) activity (21). The mechanism involves vitamin D receptor activation by its ligand (1*α*,25-dihydroxyvitamin D3) on endothelial cells, mediating anti-inflammatory and antithrombotic effects (22). The elevated DVT incidence and adverse coagulation profile observed in our deficient patients indirectly suggest that vitamin D deficiency may diminish these endothelial cytoprotective mechanisms, but this interpretation remains speculative without direct measurement of endothelial markers.

#### 4.2.3 Suppressing excessive inflammatory responses

The systemic inflammatory response triggered by surgical trauma after TKA significantly increases thrombosis risk, primarily through inflammatory mediators promoting thrombus formation via activation of coagulation factors, endothelial damage, and enhanced platelet aggregation (23). Vitamin D possesses well-documented anti-inflammatory properties that attenuate these processes; for instance, it reduces proinflammatory cytokine production, including interleukin-6 (IL-6) (24). Although this study did not assess inflammation markers, the observed hypercoagulable state in deficient patients could be partly driven by unmeasured inflammatory pathways. Previous investigations demonstrate that vitamin D deficiency correlates with elevated post-TKA levels of C-reactive protein and proinflammatory cytokines such as IL-6 and tumor necrosis factor-alpha (TNF-α) (25), which indirectly drive thrombotic progression and support our findings in a hypothesis-generating manner.

### 4.3 Clinical translation value and practice recommendations

Given the hypothesis-generating nature of these findings, definitive clinical recommendations cannot be made at this time. Nevertheless, the 2024 Endocrine Society guidelines recommend against routine vitamin D screening in healthy asymptomatic adults. However, patients undergoing major orthopedic surgery may represent a distinct population where assessment could be considered for other established benefits (e.g., bone health and muscle function). Based on the observed association and existing literature, preoperative serum 25(OH)D measurement may be considered part of a comprehensive risk assessment, with the understanding that its independent predictive value for DVT remains unproven. For deficient or insufficient patients, clinicians might consider vitamin D repletion for other established benefits (e.g., bone health and muscle function) (26). Future randomized controlled trials are needed to determine whether vitamin D supplementation specifically reduces postoperative DVT risk before any formal recommendation can be made. A multidisciplinary collaborative framework, encompassing orthopedics for risk stratification, nutrition for supplementation guidance, anesthesiology for intraoperative optimization, and nursing for postoperative monitoring, could be explored in future studies (27).

### 4.4 Research limitations

This study has several important limitations that warrant consideration.

First, the single-center retrospective design may introduce selection bias, as factors influencing vitamin D levels—such as preoperative sunlight exposure and dietary habits—were not systematically accounted for, potentially confounding the observed relationships (28).

Second, the modest sample size (*n* = 193) and low event rate (26 cases of DVT, 13.5%) limit the precision of estimates and the ability to perform extensive multivariable adjustment. The OR of 9.070 from the three-level model is based on a single DVT event in the sufficiency group, making it highly unstable. Although the binary logistic regression OR of 3.140 is more stable, the wide confidence interval (1.200–8.212) reflects the limited precision. Furthermore, the secondary multivariate model, although adequately powered in terms of EPV (13), may still be affected by sparse-data bias; Firth's penalized regression on the binary model yielded consistent results (OR = 2.918, 95% CI: 1.142–7.456), supporting the robustness of the univariate finding from the binary model. Of note, Firth regression on the three-level deficiency-vs.-sufficiency contrast would be a different modeling approach (e.g., exact logistic regression) and was not performed because of the extreme sparsity.

Third, mechanistic insights were limited by the lack of measurements for vascular endothelial markers (e.g., eNOS and VCAM-1), inflammatory cytokines (e.g., TNF-α and IL-6), or genetic polymorphisms in vitamin D metabolism pathways, omitting key biological mediators in thrombosis pathogenesis (29).

Fourth, the short postoperative follow-up period (7–10 days until discharge) failed to assess long-term VTE risk at 3–6 months, a critical gap given that short durations often obscure the chronic effects of vitamin D on health outcomes (30).

Fifth, the temporal relationship between the postoperative D-dimer measurement (day 5) and the DVT diagnosis (ultrasound between days 5–7), although largely sequential, still presents some ambiguity that limits causal interpretation. We cannot entirely exclude the possibility that some DVT events occurred before the D-dimer measurement. Finally, the absence of data on vitamin D supplementation protocols or efficacy outcomes precluded any direct conclusions about its role in reducing DVT risk (31). In addition, the small number of proximal DVT events (*n* = 5) precluded a separate subgroup analysis for clinically more relevant proximal thrombi.

## 5 Conclusion

In this hypothesis-generating analysis, preoperative serum 25-hydroxyvitamin D deficiency was associated with a higher risk of DVT following TKA in bivariate and univariate analyses. The primary three-level model produced an unstable odds ratio of 9.070 (95% CI: 1.198–68.542) for deficiency vs. sufficiency. A more stable secondary binary model (deficiency vs. non-deficiency) yielded an OR of 3.140 (95% CI: 1.200–8.212, *P* = 0.020). A secondary model adjusting for postoperative D-dimer (a potential mediator) attenuated the effect (OR = 2.576, *P* = 0.065), consistent with mediation rather than confounding. The observed alterations in D-dimer and PT suggest that vitamin D status may influence coagulation-fibrinolysis balance, but mechanistic conclusions remain speculative. The crude OR of 9.070 is based on a single DVT event in the sufficiency group and is unstable. Larger prospective multicenter studies with adequate power are urgently needed to validate these findings, establish whether preoperative vitamin D deficiency independently predicts DVT after TKA, and determine whether targeted supplementation confers thromboprophylactic benefits.

## Data Availability

The original contributions presented in the study are included in the article/Supplementary Material, further inquiries can be directed to the corresponding author.
